# Botulinum neurotoxin A mutants with enhanced ganglioside binding show improved potency and altered ganglioside selectivity

**DOI:** 10.1038/s42004-025-01569-0

**Published:** 2025-06-04

**Authors:** Geoffrey Masuyer, Andreas Rummel, Pål Stenmark

**Affiliations:** 1 Department of Biochemistry and Biophysics, Stockholm University, 10691 Stockholm, Sweden; 2 Institute of Toxicology, Hannover Medical School, 30623 Hannover, Germany

**Keywords:** X-ray crystallography, Recombinant protein therapy, Proteins

## Abstract

Botulinum neurotoxins are the causative agents of botulism, a lethal paralytic disease, but are also one of the most commonly used therapeutics for the treatment of numerous neuromuscular conditions. These toxins recognise motor nerve terminals with high specificity and affinity by using a dual binding mechanism involving gangliosides and protein receptors. The initial recognition of gangliosides is crucial for the toxins’ potency. In this study, we employed a synaptosome-binding screening strategy to identify BoNT/A mutants with enhanced ganglioside-binding which translated into improved potency. X-ray crystallography and receptor-binding assays were used to elucidate the molecular mechanisms underlying the increased affinity or altered ganglioside selectivity of these mutants. Our findings provide a basis for the development of BoNT/A variants with enhanced therapeutic potential.

## Abstract (web summary)

Botulinum neurotoxins (BoNT) are the causative agents of botulism but are also widely used to treat neurological disorders, however, the development of variants with enhanced therapeutic potential remains challenging. Here, the authors identify BoNT/A mutants with enhanced ganglioside-binding and improved potency, and elucidate the molecular mechanism underlying the increased affinity and altered ganglioside selectivity.

## Introduction

The botulinum neurotoxin family (BoNT) comprises the most toxic proteins known to man. Produced by *Clostridium botulinum*, they are the causative agent of botulism, a paralytic disease that results from the inhibition of neurotransmission at the neuromuscular junction^1^. These extremely potent toxins are also widely used to treat an ever-wider range of medical conditions, including neurological disorders such as cervical dystonia, cerebral palsy, and hemifacial spasm, as well as its well-known cosmetic applications^2^. The botulinum neurotoxin serotype A1 (BoNT/A1) is the active pharmaceutical ingredient in almost all licensed pharmaceutical products such as OnabotulinumtoxinA (Botox®), AbobotulinumtoxinA (Dysport®), IncobotulinumtoxinA (Xeomin®), PrabotulinumtoxinA (Jeuveau/Nuceiva®), LetibotulinumtoxinA (Letybo®) and DaxibotulinumtoxinA (Daxxify®).

The botulinum neurotoxin family includes seven established serotypes with multiple subtypes, which are defined by significant levels of amino acid sequence variation, with BoNT/A comprising eight subtypes (A1–A8)^3^, in addition to several recently discovered new serotypes and BoNT-like toxins^4^. They all share a common architecture made of three functional domains of 50 kDa each. The heavy chain (HC) is composed of the binding (H_C_) and translocation domains (H_N_) and is linked by a single disulphide bridge to the light chain (LC) following proteolytic activation of the toxin into its di-chain form^5^. LC is a zinc-endopeptidase responsible for cleaving one of three soluble NSF attachment protein receptors (SNARE) involved in vesicular secretion^6^.

The toxin binds specifically to the nerve terminal of cholinergic motor neurons, followed by receptor-mediated endocytosis^7^. The acidic environment of the vesicle initiates a conformational change that allows translocation of LC across the endosomal membrane and into the cytosol where it can reach its SNARE substrate, leading to inhibition of neurotransmission.

BoNT/A recognises the neuronal cell surface via a dual receptor mechanism that involves the synergistic binding of the H_C_ fragment to complex polysialo-gangliosides^7^ preassembled in a complex with synaptotagmin and the N-glycosylated synaptic vesicle glycoprotein 2 (SV2)^8–10^. Gangliosides are a family of glycosphingolipids anchored to the cell membrane that contain multiple sialic acid (*N*-acetylneuraminic acid, Neu5Ac) subunits with di-, tri- and tetrasialo-gangliosides especially present on neuronal surfaces. The complexity of gangliosides is defined by the position and number of sialic acid groups bound to Gal of the central polysaccharide chain built of Gal(β1-3)GalNAc(β1-4)Gal(β1-4)Glc^11^. The different BoNT serotypes present varying ganglioside affinities, with BoNT/A preferably adhering to the neuronally localized GT1b and GD1a than to the ubiquitously found GM1a employed by cholera toxin^12,13^.

The single ganglioside binding site (GBS) of BoNT/A1 was identified by site-directed mutagenesis. Mutation of single key residues like W1266 reduced potency of BoNT/A1 by >99% demonstrating the importance of the GBS in the mechanism of action^14^. Similar findings were obtained for BoNT/B^14^ and the related tetanus neurotoxin (TeNT)^15^ leading to the definition of the ganglioside binding motif E(D/Q)…H(K/G)…SXWY…G conserved in TeNT and BoNT/A, B, E, F, and G serotypes^16,17^. The crystal structure of BoNT/A in complex with the carbohydrate portions of GT1b and GD1a determined subsequently^18,19^ validated previous findings and provided additional details on the molecular mechanism of ganglioside recognition by clarifying the role of carbohydrate functional groups as well as of amino acids surrounding the GBS.

Recent research has shown that the binding of natural toxins to neuronal surface receptors restricts their clinical efficacy as well as their cellular targets, and that modified BoNT proteins exhibit significant potential for therapeutic applications^20,21^. Here we present results from a targeted screening using a synaptosome-binding assay that allowed the identification of key mutations in the receptor-binding site that enhanced the toxin’s cell recognition capability and potency at the neuromuscular junction. Three H_C_A mutants, Y1117V, H1253K, and Y1117V/H1253K were characterised using ganglioside-binding assays and X-ray crystallography. Y1117V and Y1117V/H1253K presented stronger affinity for GT1b and GD1a compared to the wild-type toxin, and H1253K showed an altered selectivity with a new preference for non-sialylated galactose head groups found in GD1b and GM1. These findings provide the basis for the development of engineered BoNTs with improved therapeutic efficacy and/or altered cellular selectivity.

## Results

### Synaptosome binding of H_C_A

**Fig. 1:**
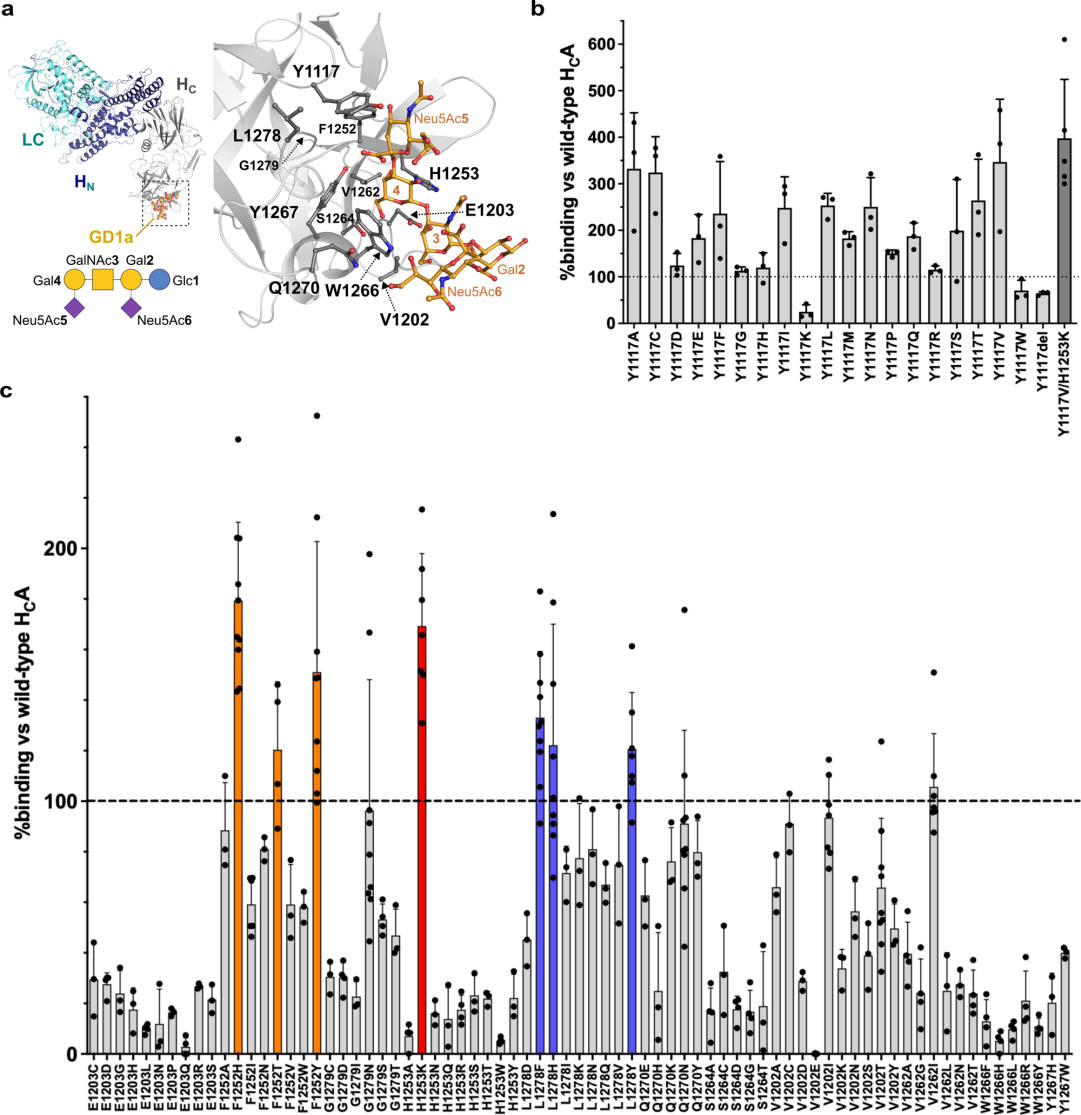
Mutant screening strategy. **a** Ganglioside binding site (GBS) of BoNT/A. Ribbon representation of the crystal structure of BoNT/A (coloured by domain with the catalytic light chain LC, in cyan, the translocation domain H_N,_ in blue, and the binding domain H_C_ in grey) bound to GD1a (in yellow), with a close view of the GBS highlighting residues screened in a synaptosome-binding assay. A glycoblock schematic of the GD1a oligosaccharide is provided. **b** Binding of ^35^S-labelled, in vitro translated H_C_A mutants to rat brain synaptosomes at 0 °C with mutations at position 1117, and **c** at further positions surrounding the binding site. Affinity of the H_C_A wild-type was set to 100% as internal reference (dotted line), error bars are standard deviation (SD) of the mean.

BoNT/A comprises a single conserved GBS built up by the motif E…H…SXWY…G (Fig. 1a). According to the H_C_A:GT1b and H_C_A:GD1a complexes, the terminal Gal4 makes CH–π interactions with W1266 while H1253 and S1264 accommodate the hydroxyl groups of Gal4^18,19^. The neighbouring Y1117 forms a hydrogen bond with the Neu5Ac moiety which moderately contributes to the binding of gangliosides GD1a and GT1b, explaining the lower binding to GM1a which lacks Neu5Ac. The homologous position 1104 in BoNT/B displays a glycine instead of tyrosine which provides more space for Neu5Ac, but makes hydrogen bonding of Neu5Ac impossible. To analyse binding of H_C_A to gangliosides incorporated in neuronal membranes, synaptosome-binding assays were conducted at 4° C which predominantly displays interaction of BoNT with gangliosides. Here, the in vitro transcribed/translated H_C_B showed a threefold superior binding than H_C_A^22^ indicating that the loss of this hydrogen bond is more than compensated by a gain of space at this area in the GBS. Although binding of the homologous mutant H_C_A Y1117G to synaptosomes was unaltered, binding of the mutant H_C_A Y1117A showed a 3-fold increase in binding to synaptosomes (Fig. 1b). To understand whether only small residues are preferred at position 1117, we systematically mutated Y1117 to all 17 remaining natural amino acids as well as deleted it and measured their binding to synaptosomes. Deletion of Y1117 and mutant Y1117W slightly decreased binding to synaptosomes, whilst mutant Y1117K severely affected it (Fig. 1b). Surprisingly, all other exchanges had a positive outcome with respect to binding. Polar amino acids like glutamate, asparagine, glutamine, serine and threonine, as well as the hydrophobic residues phenylalanine, isoleucine, leucine and methionine approximately doubled the binding affinity to synaptosomes. Cysteine, as well as alanine, caused a 3-fold increased binding while H_C_A Y1117V constituted to be the top binder with a 3.5-fold rise.

The results suggest that hydrophobic, aliphatic residues are preferred at position 1117, the smaller valine being better, but selected polar residues also being acceptable. Removal of the tyrosine hydroxyl group already caused a 2-fold gain in binding of H_C_A Y1117F despite of the loss of the hydrogen bond to Neu5Ac5, as extrapolated from the H_C_A:GD1a complex structure. The correct size/length of the side chain is an important determinant for affinity since the smallest residue glycine and the largest residue tryptophan are the second and third worst substitutions at position 1117.

Next, we analysed additional residues of the GBS motif E…H…SXWY…G for any optimization potential (Fig. 1c). Replacement of E1203 by any other polar residue clearly decreased binding, indicating that the carboxyl group and the propyl side chain are optimal. The same is true for H1253 with one exception: mutant H_C_A H1253K displayed a 1.5-fold increase in binding. An indole (W) or phenyl ring (F) could not replace the imidazole ring of H1253 demonstrating that the polar characteristics rather than the aromatic system contribute to ganglioside recognition. Interestingly, BoNT/E also presents a lysine at the homologous position 1215^23^. The core motif SXWY, strictly conserved in BoNT/A, B, E, F, G, and TeNT could not be optimized by any related amino acids, neither S1264 by small or polar residues nor W1266 and Y1267 by aromatic residues. Also, G1279 located opposite to S1264 and below Y1117, is sensitive to the addition of small side chains like hydroxymethyl as well as larger aliphatic branches as of isoleucine presumably due to the steric requirements of Gal4.

Thereafter, neighbouring positions V1202, F1252, V1262, Q1270 and L1278 were analysed (Fig. 1c). V1202 located above S1264 was mutated to 10 different residues without any positive effect on binding. Charged residues drastically diminished binding, whilst polar amino acids moderately decreased binding, and hydrophobic residues hardly affected the affinity of H_C_A towards synaptosomes. V1262 at the bottom of the GBS could only be replaced by isoleucine whereas all other aliphatic residues as well as the structurally similar residues threonine and asparagine caused a clear decrease in binding. While histidine at position 1270 was not acceptable for binding, other non-aromatic charged and polar residues were tolerated at that position. L1278, located to the right of Y1117, can be replaced by isoleucine and valine. Also, lysine, asparagine and glutamine are well tolerated, but aspartate is not. The aromatic residues histidine, phenylalanine and tyrosine increased affinity towards synaptosomes. A similar situation is observed for F1252 located between H1253 and Y1117. Aliphatic as well as polar residues caused a clear decrease in affinity with the exception of threonine. Conversely, the more polar aromatic amino acids tyrosine and histidine increased the affinity of H_C_A by 40-50%.

In a next step, the two mutations Y1117V and H1253K increasing ganglioside binding the most, were combined and analysed. The resulting H_C_A Y1117V/H1253K showed even further increased binding affinity than each individual single mutant indicating that their contributions are additive.

### Potency of BoNT/A mutants

**Fig. 2:**
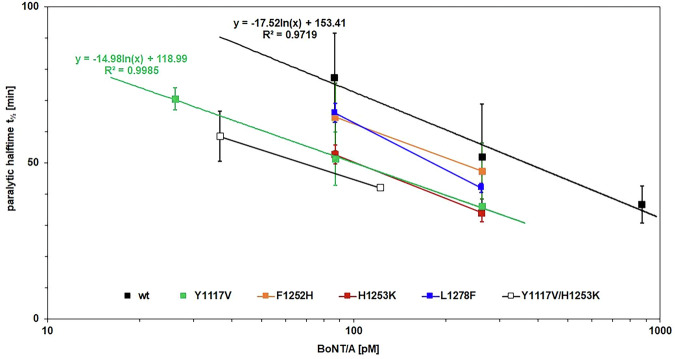
Biological activity of BoNT/A mutants with higher binding affinity. Mouse phrenic nerve (MPN) hemidiaphragm assay of 150 kDa single-chain BoNT/A wild-type and selected BoNT/A mutants based on the respective H_C_A mutants with the highest binding affinity to synaptosomes. Concentration-response curves were generated based on two to three concentrations measured in triplicates, error bars are standard deviation (SD) of the mean. The calculated mean ± SD of the paralytic halftime t_½_ was plotted against the protein concentration. Logarithmic functions were fitted and corresponding *R*^*2*^ values are provided.

To evaluate the physiological impact of H_C_A modifications, the potency of the corresponding full-length BoNT/A mutants with enhanced ganglioside-binding affinity was examined using the ex vivo mouse phrenic nerve (MPN) hemidiaphragm assay^24^ (Fig. 2). Individual dose-response curves were determined for the single mutants best-performing in the synaptosome-binding screen along with wild-type BoNT/A, to which logarithmic functions for calculating the relative potency of mutants were fitted. All single mutants showed 1.7- to 4.1-fold higher potency than the wild-type BoNT/A. Of note, the individual Y1117V and H1253K mutants performed equally and better than F1252H and L1278F and thus were combined. The resulting dual mutant Y1117V/H1253K displayed the highest potency, 5.5-fold higher than BoNT/A wild-type. Since F1252 is adjacent to H1253, we refrained from jeopardizing the positive effect of the H1253K mutation by changing its environment with the F1252H mutation. Altogether, these results support the hypothesis that improving the affinity of BoNT/A for gangliosides can result in higher potency and faster onset of action. So as to fully understand the molecular details behind this improved potency, the three best-performing candidates were analysed in detail using biochemical and biophysical assays.

### Thermodynamics of oligosaccharide recognition

**Fig. 3:**
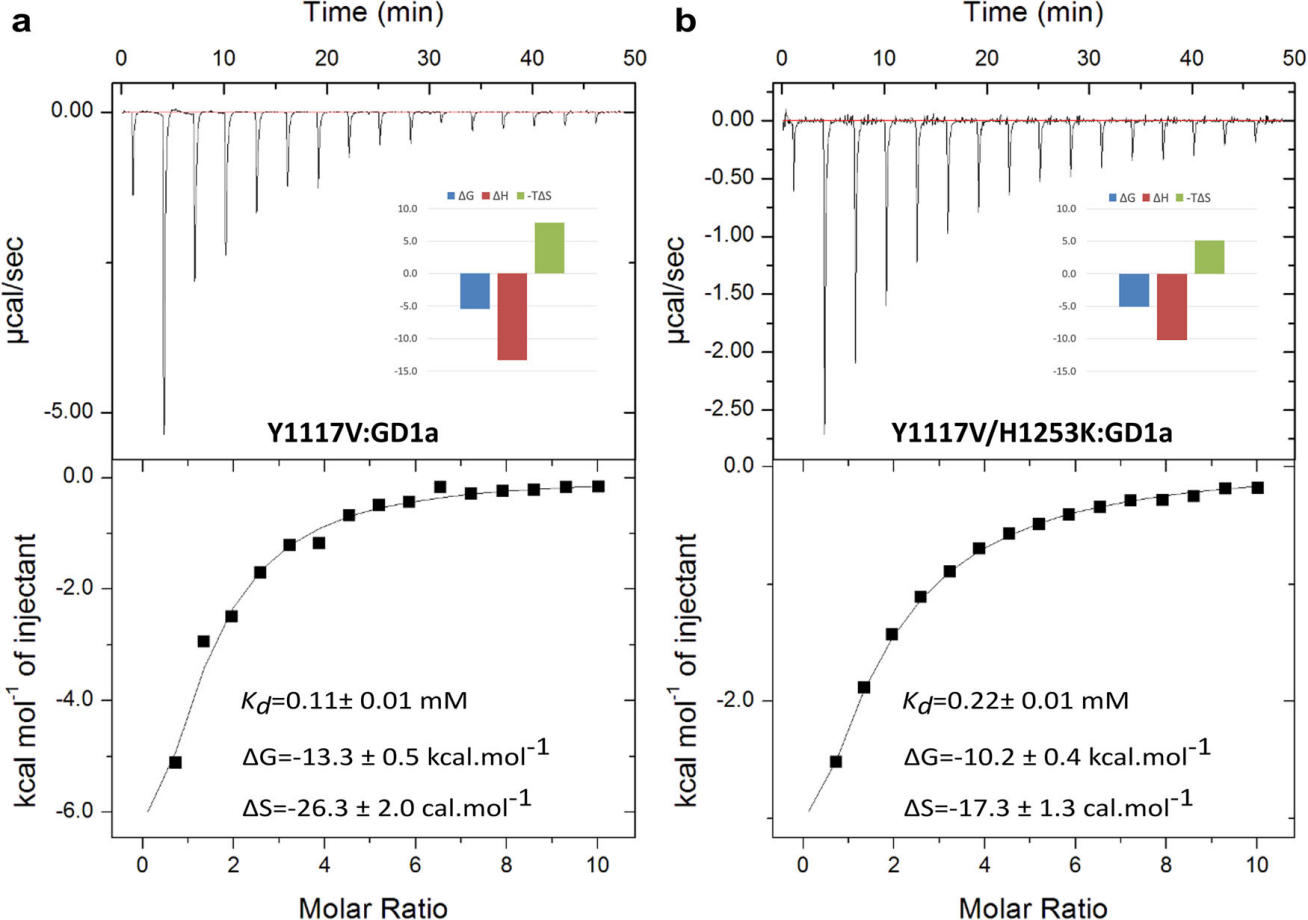
Isothermal titration calorimetry (ITC). Representative ITC measurements of the binding of GD1a by **a** H_C_A Y1117V and **b** H_C_A Y1117V/H1253K. The mean ± SD (*n* = 3) measured dissociation constant of the ligand was 0.11 ± 0.01 mM and 0.22 ± 0.01 mM, respectively. Thermodynamic profiles are also included in subpanels with mean binding free energy (ΔG), enthalpy (ΔH), and entropy (ΔS), in kcal mol^−1^.

In order to assess the biophysical binding properties of the mutants, isothermal titration calorimetry (ITC) assays were performed with the soluble ganglioside GD1a and GM1a polysaccharides. The mechanism of carbohydrate recognition by the wild-type H_C_A has been described in detail^18,19^, and showed a *K*_*D*_ of 1 mM for GD1a, and no measurable binding for GM1a. Using the same assay condition, titration of GD1a showed improved binding affinities of *K*_*D*_ = 0.11 mM and 0.22 mM for H_C_A Y1117V and Y1117V/H1253K, respectively (Fig. 3). Binding of GD1a to H_C_A H1253K could not be detected, suggesting a loss of affinity for GD1a compared to the wild-type with a *K*_*D*_ clearly above 1 mM. As for wild-type, no binding could be measured for any of the mutants when titrated with GM1a.

The thermodynamics profile of the titration reaction with GD1a is similar for Y1117V and Y1117V/H1253K mutants (Fig. 3) with a free Gibb’s energy that is predominantly driven by enthalpic contributions. Hydrophobic interfaces led by the stacking interaction of the galactose head group (Gal4) with W1266 are still likely to be the main contributors to binding, however, the increased affinity observed for the mutants may be attributed to increased electrostatic interactions created with the carbohydrate moieties (Supplementary Table 1).

### Oligosaccharide specificity

**Fig. 4:**
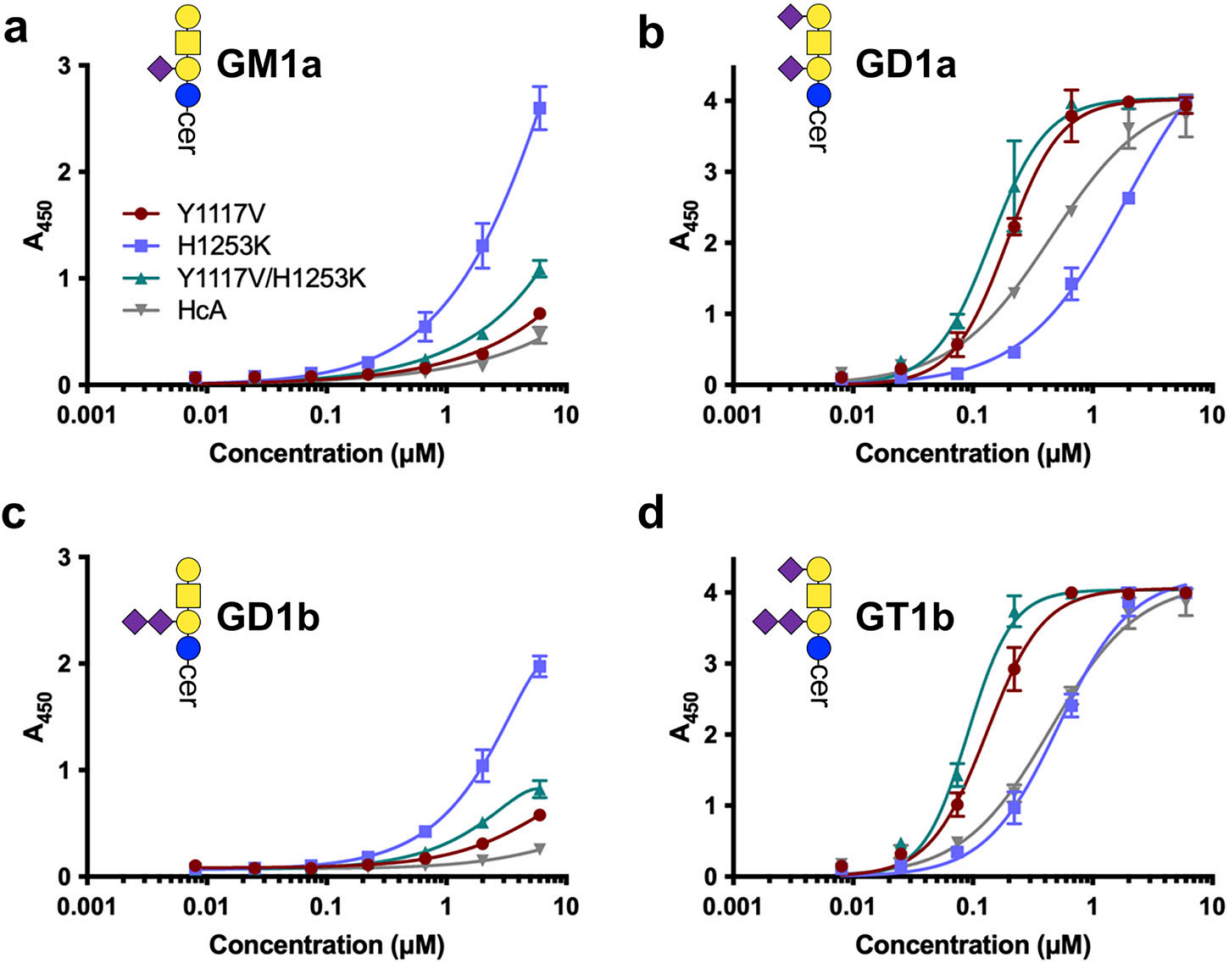
Ganglioside binding assay (ELISA). Binding of the H_C_A mutants to gangliosides **a** GM1a, **b** GD1a, **c** GD1b and **d** GT1b. EC_50_ values are summarised in Table 1. Assays were performed in triplicate with H_C_A wild-type (grey), Y1117V (red), Y1117V/H1253K (green), and H1253K (blue). Error bars are the standard error of the mean.

BoNT/A has a preference for complex gangliosides with a terminal Neu5Ac(α2-3)Gal moiety such as GD1a and GT1b^7^. The newly designed mutants were analysed to assess if they conserved the ganglioside specificity of the wild-type. For this purpose, we used a ganglioside binding ELISA, similar to the ones previously described^25,26^, in which the polysialo-gangliosides GM1a, GD1a, GD1b, and GT1b were immobilised. H_C_A Y1117V and Y1117V-H1253K presented improved binding (EC_50_ = 0.19 and 0.14 μM, respectively) to GD1a when compared to wild-type (EC_50_ = 0.45 μM). Similar results were observed with GT1b binding (Fig. 4, Table 1), whereas no significant differences were observed for GM1a and GD1b binding, although the binding by both mutants was constantly slightly above that of wild-type. These results confirm that Y1117V and Y1117V-H1253K have improved binding affinity compared to BoNT/A wild-type and also display a conserved ganglioside selectivity.

**Table 1:** Ganglioside binding ELISA analysis

	GM1a	GD1a	GD1b	GT1b
HcA (wt)	nd^a^	0.45 ± 0.06	nd	0.44 ± 0.06
HcA Y1117V	nd	0.19 ± 0.01	nd	0.13 ± 0.01
HcA Y1117V/H1253K	nd	0.14 ± 0.01	nd	0.09 ± 0.00
HcA H1253K	nd	1.9 ± 0.33	nd	0.52 ± 0.04

EC_50_ values in μM calculated from a non-linear extrapolation of the measured binding.

^a^Not determinable.

Unexpectedly, the H1253K mutant displayed an altered oligosaccharide selectivity where the affinity was reduced for GD1a and maintained for GT1b (Fig. 4, Table 1), while binding to GM1a and GD1b was improved considerably over wild-type. This single point mutation seems to favour recognition of gangliosides lacking the terminal Neu5Ac and the interaction with the Gal4, thereby changing the carbohydrate preference.

### Crystal structures of H_C_A mutants in complex with ganglioside receptors

**Fig. 5:**
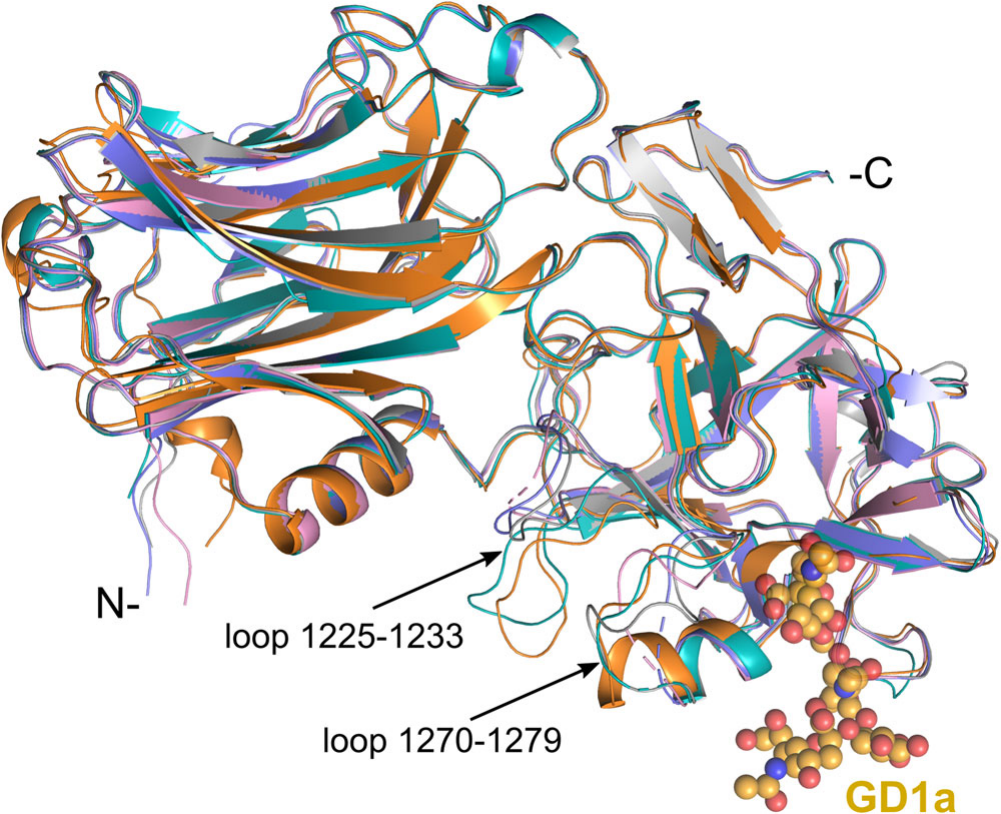
Crystal structure of the H_C_A mutants. Ribbon representation of the crystal structure of the apoprotein (orange, PDB 3BTA), GD1a-bound H_C_A (grey, PDB 5TPC), Y1117V (pink), and Y1117V/H1253K (cyan), and GM1a-bound H1253K (blue). Close-up view of the ganglioside binding site highlighting movement of loops 1270–1279 and 1225–1233.

The high-resolution crystal structures of the H_C_A mutants in complex with the polysaccharides of GD1a for Y1117V and Y1117V/H1253K, as well as GM1a for H1253K were determined (Table 2). The various constructs crystallised in different conditions and space groups, however, none of the mutations resulted in any major conformational change (Fig. 5). The most noticeable differences reside in loop 1225–1233 that is shifted considerably in the Y1117V/H1253K:GD1a structure and disordered in the Y1117V:GD1a complex. Additionally, residues 1270–1275 appeared to be somewhat disordered in all three mutant structures and could not be modelled entirely despite being visible in the wild-type BoNT/A-H_C_:GD1a structure (PDB 5TPC).

**Table 2:** X-ray crystallography: data collection and refinement statistics

	Y1117V:GD1a	Y1117V/H1253K:GD1a	H1253K:GM1a
Station	P13, PETRA III	14.1, BESSY II	I24, Diamond
Wavelength (Å)	0.976	0.918	0.968
Resolution (Å)	55–1.9 (1.94–1.90)	66–1.8 (1.84–1.80)	53–1.6 (1.63–1.60)
Space group	P2_1_2_1_2_1_	C2	C222_1_
Cell dimensions and angles	a = 73, b = 104, c = 113 Å; α = β = γ = 90°	a = 78, b = 44, c = 133 Å; α = 90, β = 96, γ = 90°	a = 73, b = 114, c = 106 Å; α = β = γ = 90°
Total/Unique reflections	413,008 69,000	135,231 39,384	422,723 59,267
Completeness (%)^a^	99.8 (99.9)	94.8 (64.9)	99.9 (99.6)
*R*_merge_	9.7 (61.8)	7.8 (24.2)	5.5 (133)
*R*_pim_	6.4 (40.7)	7.4 (21.6)	3.2 (77.2)
I/σ(I)	9.4 (2.7)	7.9 (3.8)	15.6 (2.4)
Redundancy	6.0 (6.1)	3.4 (2.5)	7.1 (7.2)
CC ½ (%)	97.7 (72.3)	98.7 (37.1)	99.9 (78.3)
*R*_*cryst*_	21.9	16.8	17.2
*R*_free_	25.9	20.7	19.7
RMSD in bond lengths (Å)	0.010	0.010	0.011
RMSD in bond angles (°)	1.46	1.59	1.56
B- factor statistics (Å^2^)			
Protein all atoms (per chain)	25.1/29.5	21.9	27.3
Ligand atoms (per chain)	36.4/42.7	53.3	84.0
Solvent atoms	30.1	28.9	38.3
Ramachandran statistics (Molprobity)			
Favoured	95.9%	95.2%	95.2%
Outliers	0.1%	0.0%	0.2%
PDB code	8RVG	8RVH	8RVI

^a^Values in parentheses refer to the highest resolution shell.

All three structures present a GBS bound by a ganglioside molecule with full occupancy (Fig. 5, Supplementary Fig. 1) with good electron density for the Neu5Ac5 (α2-3) Gal4 moiety of GD1a, and Gal4 of GM1a, as well as the central N-Acetylgalactosamine (GalNAc3). Some of the non-interacting carbohydrate moieties (Gal2) were also visible, however, Neu5Ac6 could not be modelled into the Y117V structure, and only presented very weak density in the H1253K:GM1a complex. It should also be noted that the structure of a H1253K:GD1a complex could not be obtained despite crystallisation experiments carried out in similar conditions confirming the decreased EC_50_ of H_C_A H1253K for GD1a (Fig. 4B).

### Receptor recognition

The GBS, built around the strictly conserved residues S1264, W1266, and Y1267, is common to TeNT and BoNT/A, B, E, F and G and preferentially recognises galactose. The crystal structure of the BoNT/A-H_C_:GD1a complex^19^ offered the basis of ganglioside recognition by the wild-type toxin, and a direct comparator to analyse the reason behind the properties of the novel mutants.

**Fig. 6:**
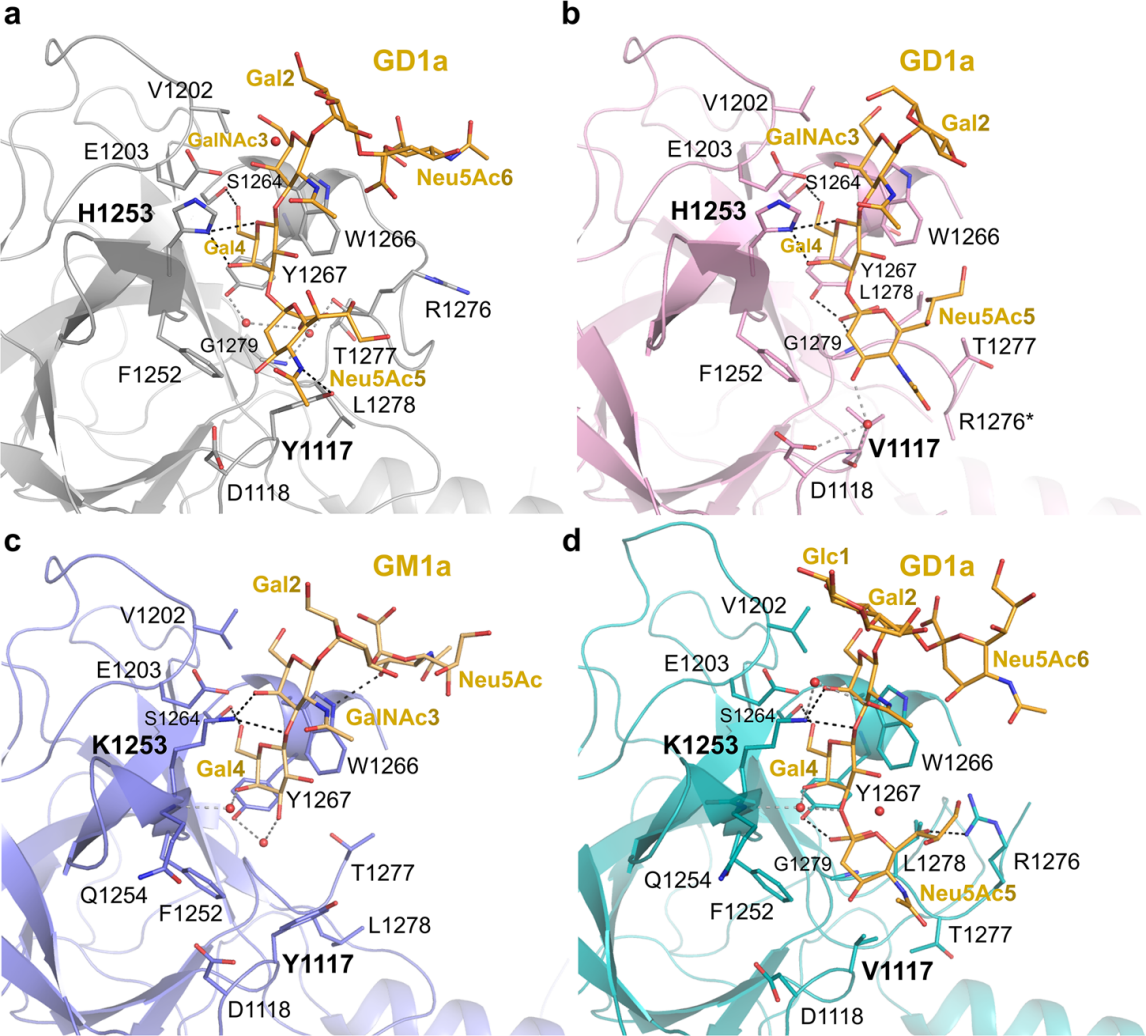
Molecular details of ganglioside binding. Ribbon representation of the crystal structure of **a** GD1a-bound H_C_A (grey, PDB 5TPC), **b** GD1a-bound Y1117V (pink), **c** GD1a-bound Y1117V/H1253K (cyan), and **d** GM1a-bound H1253K (blue). Residues involved in binding are shown in sticks, water molecules as red spheres. Hydrogen bonds are represented as dashed lines (direct bonds in black, water-mediated bounds in grey).

Careful analysis of the hydrogen-bonding network shows some variation in the number of electrostatic interactions between the native and mutant toxins (Fig. 6, Supplementary Table 1). Mutant Y1117V, while causing the loss of the Y1117V-Neu5Ac5 bond^19^, provides additional hydrogen interactions between the hydroxyl group of Y1267 and Neu5Ac5, not seen in the wild-type toxin. Furthermore, the Y1117V mutant also presents more extensive bonds to Neu5Ac5 by interacting with the main chain of G1279 (N), and making water-mediated interactions with V1117 (O) and the side chain of D1118. Mutation of H1253 to lysine also shifts the interaction of this residue towards the central GalNAc3 moiety rather than Gal4 with which it only makes water-mediated bonds via its main chain. Additionally, in the Y1117V/H1253K mutant, the more distant R1276 (NH1) forms a direct hydrogen bond with Neu5Ac5 (Fig. 6, Supplementary Table 1).

One of the major differences in the binding of H_C_A Y1117V compared to the wild-type resides in the novel hydrophobic interaction with the Neu5Ac5 group. Mutation of the bulky aromatic side chain allows the sialic acid to occupy the new hydrophobic space offered by a smaller residue (Supplementary Fig. 2; Supplementary Table 2). In addition, it also causes the repositioning of residues 1277–1279 within hydrophobic range of Neu5Ac5. It should be noticed that these residues are closer (0.5–1 Å) to Neu5Ac5 in the Y1117V mutant compared to the Y1117V/H1253K. Notably, loop 1270–1279 appears to adapt to the presence of the GD1a by presenting various flexible conformations (Fig. 5).

## Discussion

BoNT/A binds specifically to the nerve terminals at the neuromuscular junction by interacting with two surface receptors, gangliosides and the SV2 glycoprotein. Recent work suggests that while the toxin binds to SV2 with high affinity^10^, ganglioside binding occurs as a primary event providing abundance and specificity to the cellular recognition^9,19^. Gangliosides specific to neurons, GD1a and GT1b in particular, have also been shown to be essential for the toxin’s activity^27,28^. On the other hand, different neuronal tissue shows different expression patterns of complex polysialogangliosides, e.g. sensory fibres weakly express GD1a but strongly GM1a, GD1b, and GT1b whereas motor fibres are also rich in GD1a^29^. Furthermore, cholera toxin B chain, a selective marker for GM1a, only binds to large light, neurofilament-rich primary sensory neurons conveying mechanoreceptive information, but not to small dark sensory neurons being mostly nociceptive^30^. Thus, modifying the ganglioside-binding properties of BoNT/A may offer novel pharmacological benefits by modulating the neuronal cell specificity.

The ganglioside-binding site is a pocket built around the strictly conserved SxWY motif on the carboxy-terminal end of BoNT/A^14^. The difference in ganglioside selectivity observed between the various clostridial neurotoxins^31^ suggests that the mechanism of carbohydrate recognition is strongly influenced by the other surrounding residues. Previous work detailed the molecular interface between BoNT/A and GD1a^18,19^ in which, among other interactions, Neu5Ac5 was observed to form one hydrogen bond with Y1117, and Gal4 to make electrostatic interactions with H1253. This detailed map of the BoNT/A-ganglioside interaction as well as previous biochemical analysis^15^ allowed us to select key positions to screen for better receptor binders. Using a synaptosome-binding assay, we identified several mutations that significantly enhanced ganglioside affinity, in particular, mutants Y1117V, F1252H, H1253K and L1278F. The dual mutant Y1117V/H1253K, combining the best performing single mutants, was then also investigated and identified as the lead molecule with highest receptor affinity. Subsequently, the potency of these five compounds was tested in an ex vivo neuromuscular junction and all were confirmed to display a higher potency than wild-type, thereby confirming the correlation between receptor affinity and toxin potency.

Although the original screening strategy proved successful in identifying mutations with beneficial properties, synaptosomes are composed of multiple polysialo-ganglioside species. In order to understand the molecular details underlying the mutants’ enhanced properties, we analysed details of their interactions with individual gangliosides, looking at the most abundant neuronal species, i.e. GM1a, GD1a, GD1b, and GT1b.

Mutation of Y1117 to valine produced a 10-fold increase in affinity (*K*_*D*_) for the GD1a polysaccharide in solution and a 2.5-fold improvement of EC_50_ when measuring binding to immobilised gangliosides which is in good agreement with the 3.5-fold increase in synaptosomal binding. A 5-fold increase in *K*_*D*_ was observed for GD1a to the Y1117V/H1253K mutant whilst synaptosomebinding increased even 4-fold. The observed difference in *K*_*D*_ values between the double mutant Y1117V/H1253K and the single mutant Y1117V may stem from inherent variations between the assay methods, as ELISA measures relative binding under equilibrium conditions, whereas ITC provides direct thermodynamic data in solution. Replacement of the large aromatic side chain by a smaller hydrophobic residue provides a favourable interaction where the Neu5Ac5 ring can occupy the newly available sub-pocket, as illustrated by the crystal structures. Our results are in agreement with observations that a Y1117A mutant displays a stronger affinity for GD1a^32^, also offering a more open and hydrophobic area.

More surprisingly, mutation Y1117V led to conformational changes in loop 1270–1279 upon ligand binding, with residues 1276–1279 providing additional hydrophobic interaction. Although G1279 is also involved in binding of the wild-type toxin^18,19,31^, the rest of the loop takes on a different orientation in both Y1117V mutants (Supplementary Fig. 2). Despite being stabilised by interactions of the Neu5Ac5 moiety via R1276, T1277, and L1278, residues 1270–1275 were disordered, highlighting the loop’s inherent flexibility. This site therefore presents an attractive new potential in developing BoNT/A molecules with novel carbohydrate-binding properties, particularly focusing on stronger affinity for the Neu5Ac5(α2-3)Gal4 head group of GD1a and GT1b.

Single mutation of H1253 to lysine affected the ganglioside selectivity of BoNT/A by presenting a significantly higher affinity for the non-sialylated Gal4 epitope of GM1a and GD1b compared to the wild-type and Y1117V mutants. Additionally, the crystal structure of the H1253K:GM1a complex shows that the long K1253 side chain can reach the centre of the carbohydrate portion of the ganglioside via hydrogen bonds with the central GalNAc3 moiety. These results suggest that successful variation in ligand selectivity may be achieved by targeting this sub-site of the pocket, by engineering additional favourable electrostatic interactions with the GalNAc3 moiety. In the case of the single H1253K mutant, Y1117 seems to impair GD1a’s Neu5Ac5 access to the binding pocket. The combination of several mutations may provide synergistic effects to enhance the selectivity towards the galactose head group.

Overall, our results provide the molecular basis behind the enhanced binding properties of engineered BoNT/A mutants. Using a combination of biophysical methods, we demonstrate that recognition of the Neu5Ac5 motif from the neuronal GT1b and GD1a gangliosides can be improved by providing a wider hydrophobic pocket in position 1117. Furthermore, selectivity of BoNT/A towards sialyl-free Gal4 gangliosides can be altered by modifying electrostatic interactions in the upper 1253 sub-site.

Another study showed that the H_C_A Y1117A mutant^32^ bound in vitro to isolated, plastic coated GD1a and GT1b with 10- and 20-fold higher affinity, respectively, confirming our 3.3-fold increased binding of H_C_A Y1117A to synaptosomes, and presented more efficient uptake of H_C_A Y1117A into cortical neurons suggesting potentially higher potency. Here, we extensively demonstrate not only that mutations at position Y1117 can improve ganglioside-binding, but also that modifications at additional positions in the larger GBS lead to higher affinity. In particular, we identified the dual mutation Y1117V/H1253K as a high-affinity binder to GD1a, which translated into a toxin with higher potency in the mouse phrenic nerve model. Likewise, modifying the selectivity of BoNT/A towards particular gangliosides is likely to impact the toxin’s selectivity on diverse neuronal tissue, as illustrated by the natural differences between the various clostridial neurotoxins^7^. This may prove useful when considering the ganglioside composition of the target cell’s membrane.

The botulinum neurotoxins represent a unique framework for developing novel biopharmaceuticals targeting neuromuscular disorders^33^. The results provided here for the most relevant serotype A show that mutation of key residues can significantly alter the biochemical properties of BoNT/A1, and support previous findings that targeting the binding of natural toxins to neuronal surface receptors is the most effective strategy for enhancing their clinical efficacy. We therefore provide a structural template to engineer future BoNT derivatives with refined carbohydrate-binding properties that hold substantial promise for therapeutic applications.

## Methods

### Binding assay using rat brain synaptosome

Synaptosomes were obtained following the protocol described by Jones and Matus^34^. Briefly, functional synaptosomes were recovered from a Percoll gradient after homogenisation of Wistar rat brain and various centrifugation steps and finally diluted in physiological buffer (140 mM NaCl, 5 mM KCl, 1 mM MgCl2, 1 mM CaCl2, 20 mM HEPES, 10 mM glucose, 0.5% BSA, pH 7.4) with the final synaptosomal protein adjusted to a concentration of 10 mg ml^−1^. As described previously^14^, 50 kDa H_C_A-fragments (strain 62 A, protein ID AAA23262) were synthesised in vitro from pSP72 derivatives (pSPH_C_A) that were previously linearised downstream of the neurotoxins’ carboxyl-terminal codon using the reticulocyte lysate system (Promega) and L-[^35^S]methionine (555 KBq, >37 TBq mmol^−1^; Amersham Pharmacia Biotech), in a total volume of 25 µl. Binding assays were performed in triplicates, in a total volume of 100 µl of physiological buffer for 2 h at 0 °C, containing a 5 µl aliquot of the ^35^S-labelled H_C_A-fragment (reaching a final concentration of ~3 pM) and 30 µl of the synaptosome suspension. Controls were performed with samples lacking synaptosomes. After incubation, synaptosomes were collected by centrifugation (5000 × *g*; 5 min) and unbound material in the supernatant fraction was discarded. The pellet fractions were washed two times each with 50 µl of physiological buffer and incubated for 30 min at 37 °C in SDS sample buffer. Then ^35^S-H_C_A samples were analysed by SDS-PAGE, together with a 5 µl aliquot of the in vitro translation mixture to quantify the yield of radiolabelled protein. Bound ^35^S radiolabelled H_C_A-fragments were visualised using a BAS-1500 phosphor imager (Fuji Photo Film), and quantified applying the Tina 2.09f software (Raytest Isotopenmeßgeräte GmbH). Amounts of bound ^35^S-H_C_A-fragments were calculated after subtraction of the value obtained for control samples as the percentage of the total H_C_A-fragment protein in the assay, and finally expressed relative to the binding efficiency of the wild-type H_C_A-fragment (Supplementary Data 1).

### Production of recombinant 150 kDa active BoNT/A

The expression plasmid encoding the full length, 150 kDa BoNT/A wild-type with a C-terminal StrepTag (pBoNTAS) was described previously^14^ and used for site-directed mutagenesis using the GeneTailor method. The produced expression vectors encoding BoNTA wild-type and corresponding mutants were transformed into an *E. coli* K12 strain and the expression of BoNT/A was induced under biosafety level 2 containment (project number GAA A/Z 40654/3/123/3) in the *E. coli* strain M15[pREP4] (Qiagen, Hilden, Germany), following 16 h of induction at 22 °C. The single-chain neurotoxins were purified on Streptactin-Superflow (IBA GmbH, Göttingen, Germany), according to the manufacturer’s instructions, and kept in 100 mM Tris-HCl, pH 8.0 buffer. All recombinant proteins were shock frozen in liquid nitrogen, and stored at −70 °C.

### Mouse Phrenic Nerve (MPN) hemidiaphragm assay

The MPN hemidiaphragm assay was performed as described previously^22^. According to §4 Abs. 3 (killing of animals for scientific purposes, German animal protection law (TSchG)), the number of animals sacrificed by trained personnel before dissection of organs was reported yearly to the animal welfare officer of the Central Animal Laboratory and the local authority. Mice were euthanized by CO_2_ anaesthesia and subsequently exsanguinated. The phrenic nerve hemidiaphragm tissue was explanted, placed into an organ bath, and continuously stimulated at 5–25 mA with a frequency of 1 Hz and a 0.1 ms pulse duration. Isometric contractions were transformed using a force transducer and recorded with VitroDat Online software (FMI GmbH, Seeheim, Germany). The time required to decrease the amplitude to 50% of the starting value (paralytic half-time) was determined. To determine the altered neurotoxicity of mutants against the single-chain BoNT/A wild-type, concentration-response-curves, consisting of two or three data points determined minimum in triplicates, were compiled. A logarithmic function was fitted to the BoNT/A wild-type concentration-response-curve: y(BoNT/A wt; 86, 262, 873 pM) = −17.52ln(x) + 153.41; R² = 0.9719. The resulting mean paralytic half-times of the BoNT/A mutants were converted to corresponding concentrations of the BoNT/A wild-type and finally expressed as X-fold potency of BoNT/A wild-type.

### H_C_A production for biophysical analysis

The H_C_A constructs were prepared as previously described^18^. Briefly, H_C_A or its mutants were cloned into a pET-28a(+) expression vector (GenScript, NJ, USA) with an N-terminal 6× His-tag. Expression was carried out in *E. coli* BL21 (DE3) cells grown in terrific broth medium at 37 °C for approximately 3 hours and induced with a 1 mM final concentration of IPTG, overnight at 16 °C. Cells were harvested and frozen at −80 °C. Cell lysis for protein extractions was performed with an Emulsiflex-C3 (Avestin, Germany) at 20 kPsi in 0.05 M HEPES pH 7.2 with 0.2 M NaCl and 40 mM imidazole. Cell debris were spun down via ultra-centrifugation at 4 °C, 267,000 *×* *g* for 45 min. The protein was purified by affinity chromatography (HisTrap FF, GE Healthcare, Sweden), dialysis and size exclusion (Superdex200, GE Healthcare, Sweden). Samples were kept at 12 mg ml^-1^ in 0.05 M HEPES pH 7.2 with 0.2 M NaCl and 5% glycerol.

### X-ray crystallography

Samples for crystallisation were prepared by pre-incubation of the protein (10 mg ml^−1^) with 5 mM of the GD1a or 10 mM GM1a oligosaccharides (Elicityl, France). Crystals of H_C_A Y1117V:GD1a were grown with 2 μl of sample mixed with 1 μl of reservoir solution consisting of 37% v/v polyethylene glycol 300, 0.1 M phosphate citrate buffer pH 4.2, in a hanging drop set-up. For H_C_A Y1117V/H1253K:GD1a, crystals were obtained from a sitting-drop in 20% v/v polyethylene glycol 3350, 0.02 M sodium potassium phosphate, 0.1 M Bis-Tris propane pH 6.5 (PACT screen, F10, Molecular Dimensions, United Kingdom). A drop of 200 nl of sample was mixed with 100 nl of reservoir and incubated at 20 °C. Using a similar set-up, crystals of H_C_A H1253K were grown in 20% v/v polyethylene glycol 1500, 0.1 M PCPT pH 5.0 (PACT screen, C2).

Details on data collection are provided in Table 1. Complete datasets were collected from single crystals at 100 K for each complex. Raw data images were processed and scaled with Mosflm^35^, and AIMLESS^36^, using the CCP4 suite 7.0^37^. Initial molecular replacement was performed with the coordinates of the H_C_A:GD1a complex (PDB code 5TPC) to determine initial phases for structure solution in PHASER^38^. The working models were refined using REFMAC5^39^ and manually adjusted with COOT^40^. Water molecules were added at positions where Fo−Fc electron density peaks exceeded 3σ, and potential hydrogen bonds could be made. Validation was performed with MOLPROBITY^41^. Crystallographic data statistics are summarized in Table 1. Ligand interactions were analysed with LigPlot^+^^42^. All figures were drawn with PyMOL (Schrödinger, LLC, New York).

### Isothermal titration calorimetry

Samples were prepared by an additional size exclusion chromatography step (Superdex 200, GE Healthcare, Sweden) in 20 mM potassium phosphate pH 7.0, 0.15 M NaCl. Association of ganglioside polysaccharides to the binding domain of the H_C_A mutants was measured on an ITC200 (GE Healthcare, Sweden) at 25 °C and 1000 rpm. A 200 μl solution of protein (at 100 μM) was added to the cell. Binding was measured upon the addition of ganglioside (GD1a, GM1a; Elicityl, France) with 16 stepwise injections of 2.5 μL each, at a concentration of 5 mM. The first titration was set to 0.5 μl, and was subsequently deleted in the data analysis. Data (Supplementary Data 1) was analysed with the Origin software provided by the manufacturer. Titrations were performed in triplicate. The error reported for the *K*_*D*_ is the standard deviation.

### Ganglioside binding ELISA

Gangliosides GT1b, GD1b, GD1a, and GM1a were purchased from Carbosynth (Compton, UK). Gangliosides were diluted in methanol to reach a final concentration of 2.5 μg ml^−1^; 100 μL (0.25 μg) was applied to each well of 96-well PVC assay plates. After evaporation of the solvent at 21 °C (overnight), the wells were washed (3x) with 200 μL of PBS/0.1% (w/v) BSA. Nonspecific binding sites were blocked by incubation for 2 h at 21 °C in 200 μL of PBS/2% (w/v) BSA. Binding assays were performed in 100 μL of PBS/0.1% (w/v) BSA per well for 2 h at 4 °C containing the samples (serial 3-fold dilution ranging from 6 μM to 0.003 μM, with sample in triplicate wells). Following incubation, wells were washed 3x with PBS/0.1% (w/v) BSA and then incubated with an HRP-anti-His antibody (ThermoFisher #MA1-80218) at a 1:2000 dilution (100 μl/well) for 1 h at 4 °C. Finally, after three washing steps with PBS/0.1% (w/v) BSA, bound samples were detected using Ultra TMB (100 μL/well). The reaction was terminated after incubation for 5 min at 21 °C by addition of 100 μL of 1 M sulphuric acid. Absorbance at 450 nm was measured with a Tecan Infinite 200 (Männedorf, Switzerland). Assays were performed in triplicates. Results (Supplementary Data 1) were analysed with Prism (GraphPad, La Jolla, CA, USA), using a non-linear binding fit.

## Supplementary information

Supplementary material

Description of Additional Supplementary Files

Supplementary Data 1

## Data Availability

The atomic coordinates and structure factors (codes 8RVG, 8RVH, and 8RVI) have been deposited in the Protein Data Bank (http://wwpdb.org). Data from mutation screening and ganglioside-binding are provided in Supplementary Data 1. Other datasets generated during the current study are available from the corresponding author upon reasonable request.
