# Supplementary material for: Botulinum neurotoxin A mutants with enhanced ganglioside binding show improved potency and altered ganglioside selectivity
Source: Commun Chem. 2025 Jun 4;8:171. doi: 10.1038/s42004-025-01569-0 (PMC12137935; doi:10.1038/s42004-025-01569-0)
Supplement: Supplementary file 1 — Supplementary material [file 42004_2025_1569_MOESM1_ESM.pdf]

Supplementary Information

Supplementary Fig. 1. Ganglioside binding. Ribbon representation of the crystal structure of **(a)** GD1a-bound Y1117V (pink), **(b)** GD1a-bound Y1117V/H1253K (cyan), and **(c-d)** GM1a-bound H1253K (blue). Electron density maps (*2Fo-Fc*) represented in blue mesh around the mutated residue and ganglioside (at 1σ in a-c, and 0.75σ in d).

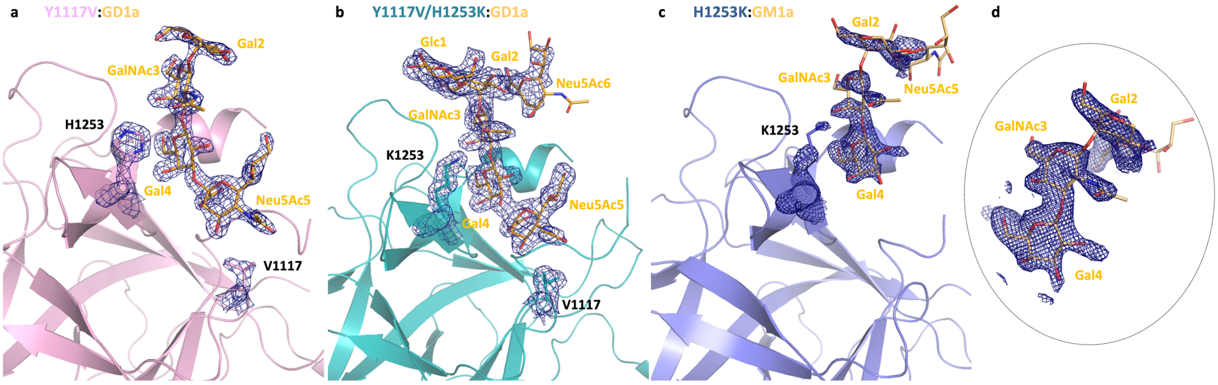

Supplementary Fig. 2. Comparison of ganglioside binding between H_CA mutants. Ribbon representation of the crystal structure of **(a)** GD1a-bound H_CA (grey, PDB 5TPC) superposed with GD1a-bound Y1117V (pink), **(b)** GD1a-bound H_CA (grey, PDB 5TPC) superposed with GD1a-bound Y1117V/H1253K (cyan), and **(c)** GD1a-bound H_CA (grey, PDB 5TPC) superposed with GM1a-bound H1253K (blue).

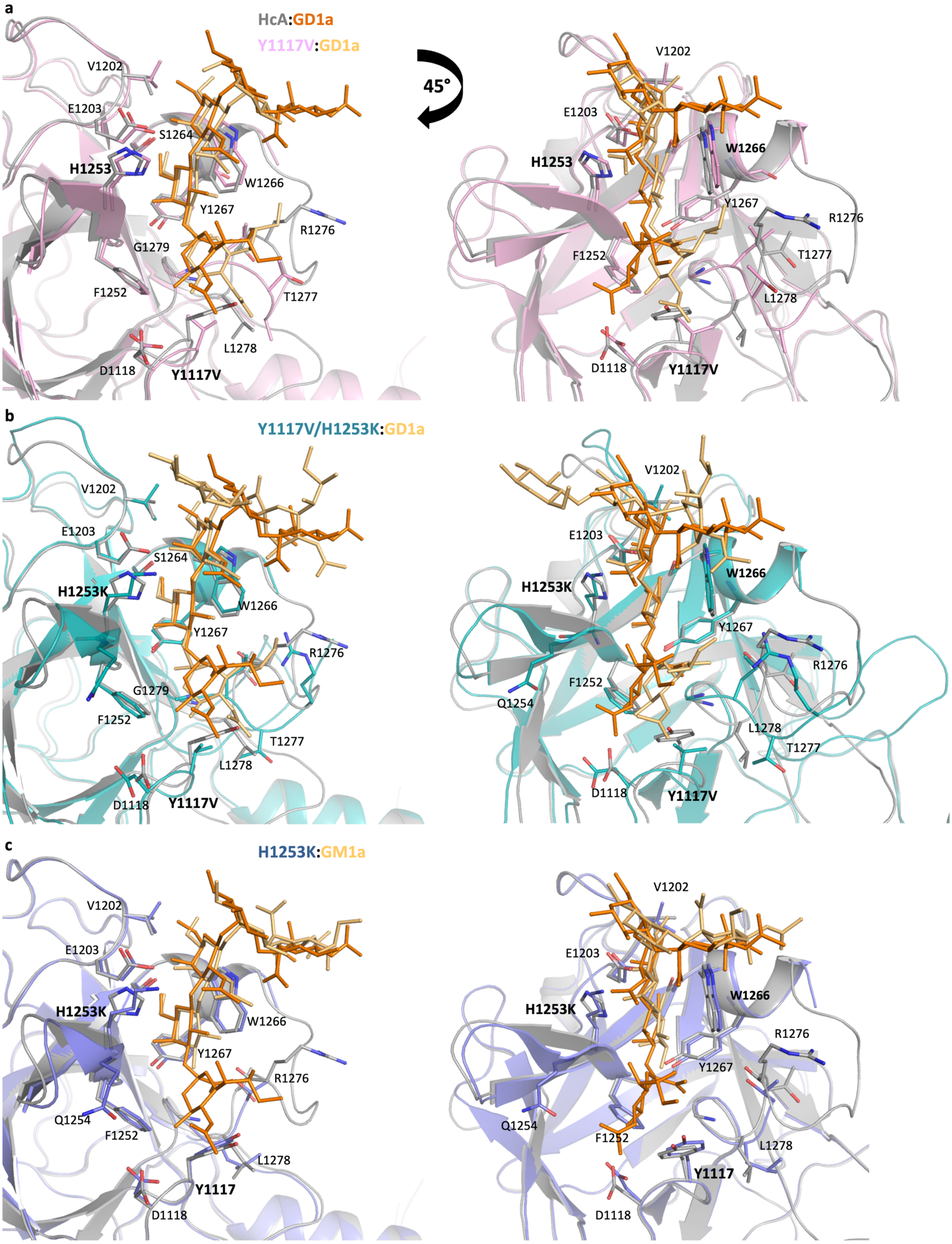
a
HcA:GD1a
Y1117V:GD1a
45°
V1202
E1203
S1264
H1253
W1266
Y1267
R1276
G1279
F1252
T1277
L1278
D1118
Y1117V
b
Y1117V/H1253K:GD1a
H1253K
Q1254
c
H1253K:GM1a
Y1117

Supplementary Table 1. Summary of hydrogen bonds between H_CA mutants and their bound polysaccharides. (blue area corresponds to water-mediated interactions; greyed area to the lack of Neu5Ac5 in GM1a)

Ligand		**HcA:GD1a**		**Y1117V:GD1a**		**Y1117V/H1253K:GD1a**		**H1253K:GM1a**	
Residue	Atom	Residue	Distance	Residue	Distance	Residue	Distance	Residue	Distance
Nga3	O3					K1253 NZ	2.9	K1253 NZ	3.3
Nga3	O4					K1253 NZ	2.9	K1253 NZ	2.7
Nga3	O7					Wat-K1253 NZ	3.0		
Gal4	O3					Wat-Q1254 N	2.7	Wat-Y1267 N	2.7
Gal4	O4					Wat-Q1254 N	2.9	Wat-Q1254 N	3.0
Gal4	O4	H1253 ND1	2.7	H1253 ND1	2.6				
Gal4	O5	H1253 ND1	3.2	H1253 ND1	3.1				
Gal4	O6	S1264	2.8	S1264 OG	2.7	S1264 OG	2.7	S1264 OG	2.6
Neu5Ac5	O1A			Y1267 OH	2.6	Y1267 OH	2.6		
Neu5Ac5	O1A			G1279 N	2.9				
Neu5Ac5	01B	Wat-G1279 N	2.8						
Neu5Ac5	01B	Wat-R1276 O	2.8						
Neu5Ac5	01B	Wat-Y1267	2.5						
Neu5Ac5	O4			Wat-D1118 OD1	2.6				
Neu5Ac5	O4			Wat-V1117 O	2.6				
Neu5Ac5	N5	Y1117 OH	2.9						
Neu5Ac5	O7					R1276 NH1	3.0		
Neu5Ac6								W1266 NE1	3.2

Supplementary Table 2. Summary of hydrophobic interactions between H_CA mutants and their bound polysaccharides. (greyed area corresponds to the lack of Neu5Ac5 in GM1a)

Ligand	**HcA:GD1a**	**Y1117V:GD1a**	**Y1117V/H1253K:GD1a**	**H1253K:GM1a**
GalNAc3	V1202			V1202
Gal4	E1203	E1203	E1203	
Gal4	W1266	W1266	W1266	W1266
Gal4	Y1267			
Neu5Ac5		V1117	V1117	
Neu5Ac5	F1252	F1252	F1252	
Neu5Ac5		T1277		
Neu5Ac5		L1278		
Neu5Ac5			G1279	
Neu5Ac6				W1266
