# Supplementary material for: Botulinum neurotoxin A mutants with enhanced ganglioside binding show improved potency and altered ganglioside selectivity
Source: Commun Chem. 2025 Jun 4;8:171. doi: 10.1038/s42004-025-01569-0 (PMC12137935; doi:10.1038/s42004-025-01569-0)
Supplement: Supplementary file 2 — Description of Additional Supplementary Files [file 42004_2025_1569_MOESM2_ESM.pdf]

Description of Additional Supplementary Files

File name: Supplementary Data 1

Description: Data files from experiments, including mutant screening assay (Figure 1), and ganglioside-binding assays (Figure 3 and 4).
